# Mobile phone dependency and sleep quality in college students during COVID-19 outbreak: the mediating role of bedtime procrastination and fear of missing out

**DOI:** 10.1186/s12889-023-16061-4

**Published:** 2023-06-21

**Authors:** Tao Huang, Yiting Liu, Teck Cheng Tan, Dong Wang, Kefeng Zheng, Wenxi Liu

**Affiliations:** 1 Department of Physical Education, Shanghai Jiao Tong University, Shanghai, China; 2 School of Kinesiology and Health, Capital University of Physical Education and Sports, Beijing, China

**Keywords:** Mobile phone dependency, Sleep quality, Bedtime procrastination, Fear of missing out, College students

## Abstract

**Objective:**

The purpose of this study was to investigate the associations between mobile phone dependency, bedtime procrastination, FoMO, and sleep quality among college students during the COVID-19 outbreak. Specifically, we examined whether bedtime procrastination and FoMO mediate the relationship between mobile phone dependency and sleep quality.

**Methods:**

A total of 881 college students completed an online survey in May 2022 in Shanghai, China. Mobile Phone Involvement Questionnaire, Bedtime Procrastination Scale, Fear of Missing Out Scale and Pittsburgh Sleep Quality Index were used to assess mobile phone dependency, bedtime procrastination, fear of missing out, and sleep quality, respectively. Multiple linear regression and mediation analysis were conducted.

**Results:**

The correlation analyses indicated mobile phone dependency was positively associated with fear of missing out, bedtime procrastination, and poor sleep quality among college students. The structural equation modeling analyses revealed that mobile phone dependency had significant indirect effects on sleep quality through bedtime procrastination (indirect effect: 0.030, 95%CI: 0.022–0.041) and fear of missing out (indirect effect: 0.013, 95%CI: 0.003–0.023).

**Conclusion:**

The findings indicated that bedtime procrastination and fear of missing out are mediators mediating the relationship between mobile phone dependency with sleep quality. Bedtime procrastination and fear of missing out should be considered as potential intervention targets for reducing mobile phone dependency and improving sleep quality in college students.

## Introduction

The emergence of mobile phone technology has transformed the way individuals communicate, work, and entertain themselves. While the increased use of mobile phones has numerous benefits, such as increased connectivity, it also has potential negative effects on mental health and well-being [1]. According to the 50th China Internet Development Statistics Report released by China Internet Network Information Center (2022) (CNNIC), as of June 2022, the size of mobile phone users in China was 1.05 billion. The proportion of Internet users using mobile phones to access the Internet was 99.6%. Among them, the age group of 20–29 years old accounted for 17.2% [2]. One area of concern is the impact of mobile phone use on sleep quality, particularly among college students.

College students are a vulnerable population, as they often have irregular sleep patterns due to academic demands, social activities, and part-time jobs [3]. Furthermore, the COVID-19 pandemic has disrupted their daily routines, leading to increased stress, anxiety, and mobile phone use [4]. During the pandemic, the education system has undergone a transformation, with distance learning becoming the norm for many college students. This shift has led to an increase in mobile phone usage, as college students rely on mobile phones to participate in online classes, communicate with peers, and access academic resources. Mobile phone dependency, defined as the excessive use of mobile phones that leads to negative consequences such as addiction, has become a common phenomenon among college students [5]. Previous studies have shown that excessive mobile phone use is associated with poor sleep quality among college students, which can negatively impact their academic performance, mental health, and overall well-being [6, 7]. Reports from recent studies including large sample size have revealed that over 50% of Chinese college students were mobile phone dependency [8, 9].

However, the mechanisms underlying the relationship between mobile phone use and poor sleep quality are not well understood. Recent research suggested that bedtime procrastination and fear of missing out (FoMO) may play a mediating role in this relationship [10]. To address this gap, the present study aimed to investigate the mediating roles of bedtime procrastination and fear of missing out (FoMO) in the relationship between mobile phone dependency and poor sleep quality among college students during the COVID-19 outbreak.

Bedtime procrastination, which refers to delaying the time of going to bed, has been identified as a factor that contributes to poor sleep quality [11]. The use of mobile phones before bedtime has been found to be associated with bedtime procrastination [12, 13]. Meanwhile, a meta-analysis indicated that bedtime procrastination was associated with poorer sleep quality [14]. Therefore, it is possible that bedtime procrastination mediates the relationship between mobile phone dependency and poor sleep quality among college students.

Furthermore, fear of missing out, which refers to the anxiety that people experience when they believe that they are missing out on social experiences, has been identified as a factor that contributes to excessive use of mobile phones [15]. Previous studies have demonstrated that social media use and problematic mobile phone use were associated with higher level of fear of missing out [16, 17]. Meanwhile, fear of missing out was associated with poorer sleep quality [18, 19]. Therefore, it is possible that FoMO mediates the relationship between mobile phone dependency and poor sleep quality among college students.

Understanding the mediating roles of bedtime procrastination and FoMO in the relationship between mobile phone dependency and poor sleep quality among college students may have important implications for the development of interventions to reduce mobile phone dependency and improve sleep quality in this population. Therefore, the present study aimed to examine the associations between mobile phone dependency, bedtime procrastination, FoMO, and sleep quality, and to investigate the mediating roles of bedtime procrastination and FoMO in the relationship between mobile phone dependency and poor sleep quality among college students during the COVID-19 outbreak. It was hypothesized that (a) mobile phone dependency, bedtime procrastination, FoMO would be associated with sleep quality; (b) bedtime procrastination would mediate the association between mobile phone dependency and sleep quality; (c) FoMO would mediate the association between mobile phone dependency and sleep quality. The findings of present study will provide a deeper understanding of the mechanisms underlying the relationship between mobile phone dependency and poor sleep quality. Identifying mediating factors can inform future studies better understand why mobile phone dependency can negatively impact sleep quality and develop effective interventions.

## Methods

### Participants

An online survey was conducted in several universities in Shanghai to collect data during the Covid-19 outbreak in May, 2022. Undergraduate and master students who volunteered to participate in the study were invited to complete the survey posted online (wjx.cn). The questionnaires in this survey were completed in Chinese version, which may nearly 10 min to fill in. A total of 881 respondents completed the online questionnaires. 862 participants (mean age = 20.51 ± 2.58, female 45%) provided valid responses and included in the analyses. Ethical approval was obtained from the Institional Review Board for Human Research Protections at Shanghai Jiao Tong University.

### Measures

#### Mobile phone involvement questionnaire (MPIQ)

The Mobile Phone Involvement Questionnaire was compiled by Walsh [20], which has been translated in the Chinese version and validated (Cronbach’s α = 0.849) in the university population by Lin et al. [21]. The MPIQ contains 8 items related to the perception and behavior towards mobile phone. Each item was scored based on a 7-Likert scale and the total score ranges from 8 to 56 points. A higher total score indicates the greater mobile phone dependency, and 32 points was used as cut-off points for distinguishing the mobile phone dependent.

#### Bedtime Procrastination Scale (BPS)

The Bedtime Procrastination Scale was compiled by Kroese [11] which has been used for assessing bedtime procrastination behavior of the participants. The Chinese version of the BPS has been validated among Chinese university students (Cronbach’s α = 0.91) [22]. The BPS contains 9 items related to the contexts associated with bedtime procrastination behavior and each item was scored based on a 5-point Likert scale, notably, item 2, 3, 7 and 9 were reverse scored. The total score ranges from 9 to 45 points, which the higher total score indicates more severe bedtime procrastination.

#### Fear of missing out Scale (FoMOs)

The Fear of Missing Out Scale was compiled by Przybylski [15] and the Chinese version [23] (Cronbach’s α = 0.72) has been widely used for measuring the level of fear of missing out in online and offline contexts. The Chinese version of FoMOs contains 8 items and each item is scored based on a 5-point Likert scale (1 = totally not true for me; 5 = extremely true for me). And the 8-item FoMO was consisted of two factors including fear of missing out on information and context, and each factors had 4 items. For example, one of the items for fear of missing out on information is “I fear others have more rewarding experiences than me.” And one of the items for fear of missing out on context is “It bothers me when I miss an opportunity to meet up with friends.” The total score ranges from 8 to 40 points and a higher score indicates the higher level of fear for missing out.

#### Pittsburgh Sleep Quality Index (PSQI)

The Pittsburgh Sleep Quality Index was compiled by Buysee [24], which has been widely used for assessing the sleep quality of the participants in the last one month. The Chinese version has been translated by Lu et al. [25]. The 19-item PSQI scale was stratified to 7 components including sleep quality, sleep latency, sleep duration, habitual sleep efficiency, sleep disturbances, use of sleeping medication, and daytime dysfunction. The score of each component ranges from 0 to 3 points and the total score ranges from 0 to 21 points. The higher total scores indicate the poorer the sleep quality.

### Statistical analyses

Potential differences on participant characteristics and study outcomes between female students and male students were evaluated by independent sample T-test. Correlations among the main variables were analyzed using Pearson correlations. Structural equation modeling was used to assess the mediating effects of fear of missing out and bedtime procrastination. The statistical analysis was performed using a commercial statistical package SPSS. The null hypothesis was rejected for two-sided values of P < 0.05.

## Results

### Descriptive statistics and correlation analysis

As presented in Table 1, the mean age of participants was 20.51 ± 2.58 years old. The mean score of mobile phone dependency, bedtime procrastination, fear of missing out and sleep quality respectively was 33.79 ± 8.32, 30.10 ± 7.09, 23.85 ± 6.14, 5.58 ± 2.79. For mobile phone dependency, more than half of all participants met the criteria for mobile phone dependent (63.7%). For fear of missing out, most participants had a mild to moderate level of fear of missing out (85.6%). For bedtime procrastination, more than half of the participants had some degree of bedtime procrastination (65.7%). For sleep quality, more than half of the participants achieved very good sleep quality (51.5%). Furthermore, with the exception of mobile phone dependency, the other three variables were statistically different between male and female (P < 0.05).

**Table 1:** Descriptive statistics for study outcomes (N = 862)

		Male (%)	Female (%)	Total (%)	*p*
Mobile phone dependency	Non-dependents Dependents Mean ± SD	124 (39.9) 285 (60.1) 33.33 ± 0.41	189 (32.0) 264 (68.0) 34.35 ± 0.378	313 (36.3) 549 (63.7) 33.79 ± 8.32	0.07
Fear of missing out	< 16 points 16–24 points 25–32 points > 32 points Mean ± SD	45 (9.5) 238 (50.2) 161 (34.0) 30 (6.3) 23.44 ± 0.30	27 (7.0) 167 (43.0) 172 (44.3) 22 (5.7) 24.35 ± 0.29	72 (8.4) 405 (47.0) 333 (38.6) 52 (6.0) 23.85 ± 6.14	0.03
Bedtime procrastination	< 18 points 18–27 points 28–36 points > 36 points Mean ± SD	26 (5.5) 153 (32.3) 221 (46.6) 74 (15.6) 29.63 ± 0.32	18 (4.6) 99 (25.5) 196 (50.5) 75 (19.3) 30.67 ± 0.36	44 (5.1) 252 (29.2) 417 (48.4) 149 (17.3) 30.10 ± 7.09	0.03
Sleep quality	Very good A little good A little bad Very bad Mean ± SD	250 (52.7) 205 (43.2) 18 (3.8) 1 (0.2) 5.39 ± 0.13	194 (50.0) 166 (42.8) 28 (7.2) 0 (0.0) 5.81 ± 0.14	444 (51.5) 371 (43.0) 46 (5.3) 1 (0.1) 5.58 ± 2.79	0.03

*Note*: ^*^p < 0.05, ^***^p < 0.001 (Two-tailed test)

The correlation among the following 4 variables, MPIQ (mobile phone dependency), FoMOs (fear of missing out), BPS (bedtime procrastination) and PSQI (sleep quality), was analyzed by Pearson related analysis. As showed in Table 2, there were positive correlation between MPIQ and FoMOs (r = 0.441, P < 0.01), MPIQ and BPS (r = 0.306, P < 0.01), MPIQ and PSQI (r = 0.291, P < 0.01), respectively. The results indicated mobile phone dependency was separately associated with fear of missing out, bedtime procrastination, and sleep quality. FoMOs was positively correlated with BPS (r = 0.174, P < 0.01) and PSQI (r = 0.209, P < 0.01), suggesting severe bedtime procrastination and poor sleep quality were related to higher level of fear of missing out. In addition, the positive correlation was also found between BPS and PSQI (r = 0.360, P < 0.01), which indicated bedtime procrastination was associated with poor sleep quality.

**Table 2:** Pearson correlation analysis

Variable	MPQI	FoMOs	BPS	PSIQ
MPIQ	1			
FoMOs	0.441^**^	1		
BPS	0.306^**^	0.174^**^	1	
PSQI	0.291^**^	0.209^**^	0.360^**^	1

*Note*: ^**^p < 0.01 (Two-tailed test)

### Testing for mediation model

**Fig. 1:**
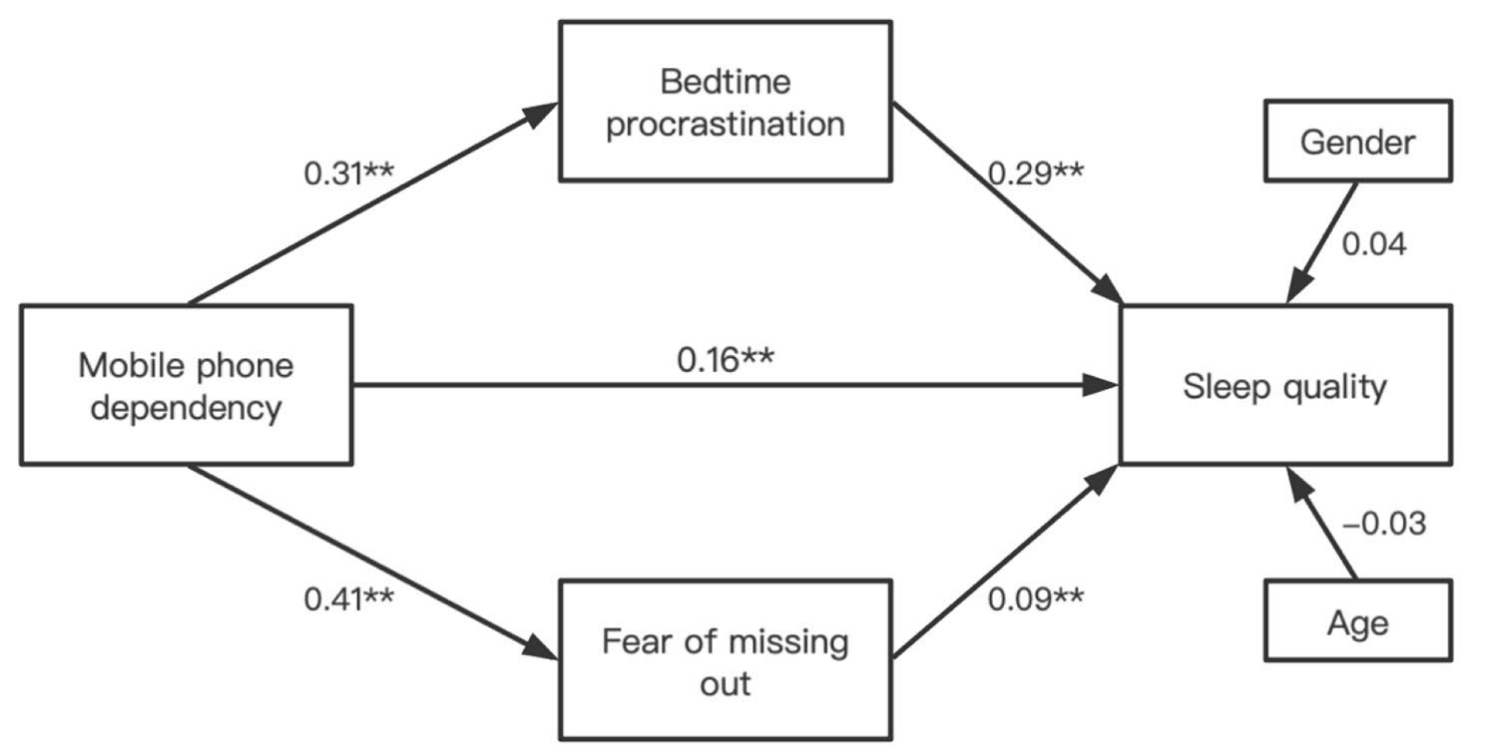
The structural equation modeling of this study. *Note*: ^**^: p < 0.01

The results of mediation model was shown in Fig. 1. After controlling for the effects of age and sex, the structural equation model resulted in the following model fitting indices: χ2/df = 6.71, goodness-of-fit index (GFI) = 0.98, adjusted goodness-of-fit index (AGFI) = 0.95, comparative fit index (CFI) = 0.90, incremental fit index(IFI)=0.90, root mean square error of approximation (RMSEA) = 0.08, which indicated the model fitting is acceptable [26]. To examine the mediated effects associated with mobile phone dependency and sleep quality, using bias-corrected bootstrap 95% confidence intervals in 5000 samples. Bootstrap analysis results indicated that confidence intervals for the mediated effects of bedtime procrastination did not contain zero and the same result appeared in the mediated effects of fear of missing out. Therefore, bedtime procrastination and fear of missing out both mediated the link between mobile phone dependency and sleep quality, which indicated that mobile phone dependency had a significant indirect effect on sleep quality through bedtime procrastination and fear of missing out.

**Table 3:** Testing for mediation model

Pathway		Standardized Effect Size	95% CI
Direct effects			
Mobile phone dependency	→Bedtime procrastination	0.31	0.24, 0.37
	→ Fear of missing out	0.41	0.34, 0.48
	→ Sleep quality	0.16	0.10, 0.24
Indirect effects			
Mobile phone dependency	→ Bedtime procrastination→ Sleep quality	0.09	0.07, 0.12
Mobile phone dependency	→ Fear of missing out→ Sleep quality	0.04	0.01, 0.07
Total effect			
Mobile phone dependency	→ Sleep quality	0.29	0.23, 0.35

As showed in Table 3, the results revealed that mobile phone had a significant indirect effect on sleep quality through bedtime procrastination (indirect effect: 0.09, 95%CI: 0.07 ~ 0.12). Meanwhile, mobile phone also had a significant indirect effect on sleep quality through fear of missing out (indirect effect: 0.04, 95%CI: 0.01 ~ 0.07). The total mediated effect from mobile phone dependency to sleep quality was statistically significant (total mediated effect: 0.13), while the direct path from mobile phone dependency to sleep quality was significant as well (total effect: 0.29, 95%CI: 0.23 ~ 0.35). Thus, bedtime procrastination and fear of missing out played mediating role in the relationships on the association between mobile phone dependency and sleep quality.

## Discussion

The present study examined the associations between mobile phone dependency, bedtime procrastination, FoMO, and sleep quality among college students. Moreover, the present study also examined the mediation effects of mobile phone dependency on sleep quality via bedtime procrastination and FoMO. Overall, the findings were in line with previous studies regarding the associations between mobile phone dependency and FoMO, bedtime procrastination, and sleep quality. Furthermore, bedtime procrastination and FoMO were observed to mediate the relationship between mobile phone dependency and sleep quality.

The mobile phone dependency was positively correlated with poor sleep quality. Consistent with previous studies, college students with high level of mobile phone dependency were more likely to have poor sleep quality [7, 27−29]. The correlation coefficient between mobile phone dependency and sleep quality (r = 0.29) was similar to a recent meta-analysis result (r = 0.28) [30]. Commonly, the excessive mobile phone use before bedtime would postpone or even replace sleep time [31]. And considering the context of the Covid-19 epidemic, the replacement of sleep time with mobile phone screen may exacerbate the negative effects [32]. Also, higher level of mobile phone dependency may result in a higher level of psychological stress or arousal, which may disturb the sleep [33, 34].

The mobile phone dependency was positively associated with bedtime procrastination and FoMO, which indicated that college students who had higher level of mobile phone dependency may experience greater bedtime procrastination and FoMO. In line with the findings from a recent meta-analysis, individuals who spent more time on electronic media use were more likely to have greater level of bedtime procrastination [35]. Moreover, the present study also revealed that the bedtime procrastination was positively associated with poor sleep quality, which is consistent with the meta-analysis findings [35]. In addition, consistent with previous study [18], mobile phone dependency was positively associated with FoMO, which indicated that college students who had higher level of mobile phone dependency may experience greater level of FoMO. And during the epidemic, college students were tended to rely on using social media via mobile phones to connect with others. When there is a gap between the restrictions of one’s own area and the freedom of others, this can create jealousy and trigger fear of missing out [36, 37]. Number of studies revealed that mobile phone addiction usually associated with social media addiction since social media use is primarily accessed via mobile phones [38, 39]. It is prevalent using social media via mobile phones before bedtime among college students, and the study findings indicated that college students with higher level of mobile phone dependency may stay prolonged time on social media which cause greater level of FoMO.

The present study findings supported the mediation effects of bedtime procrastination between mobile phone dependency and sleep quality. In the context of mobile phone use, it is possible that individuals who are dependent on their mobile phones may engage in excessive use before bedtime, which may contribute to bedtime procrastination and, in turn, poor sleep quality. The mediating role of bedtime procrastination found in this study is consistent with previous research that has linked bedtime procrastination to poor sleep quality [40, 41]. College students who had greater level of mobile phone dependency may have difficulties to stop using mobile phones before bedtime (e.g., pleasure-seeking activities via mobile phones, chatting online, playing games), which may cause higher level of bedtime procrastination and lead to poorer sleep quality such as shorter sleep duration, longer sleep latency, or even insomnia [33, 42]. Moreover, in terms of gratification theory of internet use, college students inclined to use their mobile phones to get gratification and pleasure after a long school-day for relaxing themselves [43]. Bedtime procrastination as a relatively new concept related to health behavior, the present study suggested the mediating role of bedtime procrastination between mobile phone dependency and sleep quality. The indirect effects of mobile phone dependency on sleep quality through bedtime procrastination provided insights and direction for future study investigating the correlates of mobile phone addition and sleep quality.

The present study supported that mobile phone dependency had an indirect effect on sleep quality through FoMO. In the context of mobile phone use, it is possible that individuals who are dependent on their mobile phones may feel a constant need to check their phones for fear of missing out on important information or social interactions. This constant need to check their phones may disrupt their sleep and contribute to poor sleep quality. The mediating role of FoMO found in this study is consistent with previous research that has linked FoMO to poor sleep quality [18, 44]. College students with mobile phone dependency are more likely to experience higher level of FoMO, in turn, lead to poor sleep quality. Similarly, Li et al. [18] in a cross-sectional study found that individuals who had higher level of negative affect were more prone to experience higher levels of FoMO and suffer smartphone addiction, eventually, led to poor sleep quality. FoMO was described as a negative psychological state under the social media context, which individuals would continuously worry about missing online information, messages, and social interactions [45]. In China, the most popular social media mobile phone application is called WeChat, which facilities people’s daily life, such as messaging, making video calls, chatting online, etc. College students are the “digital natives” who live depend on such social media and stay connected with others. Study evidence has shown the strong correlation between smartphone addiction and FoMO, as the information or message were kind of updated and exchanged constantly, thus, individuals with high level of mobile phone dependency were more likely to frequently checking social media via their mobile phones to avoid of missing out online information, thereby, aggravated the level of FoMO and affecting sleep quality eventually [46].

Notably, the present study also observed significant association between bedtime procrastination and FoMO. Bedtime procrastination was considered as health-related behavior, whereas FoMO was defined as health-related psychological state. The present study supported both were mediators mediating the association between mobile phone dependency and sleep quality. Similar findings were also found in one study, which reported that FoMO mediated the link between social media use and sleep outcomes via behavioral and psychological path [47]. The late-night social media use aggravated FoMO which led to delayed sleep time, meanwhile, on the cognitive level, social media use at night with FoMO increased cognitive arousal before bedtime and further delayed sleep onset.

The finding that bedtime procrastination and FOMO partially mediate the relationship between mobile phone dependency and sleep quality suggests that interventions targeting these factors may be effective in improving sleep quality in college students who are dependent on their mobile phones. For example, interventions that promote healthy sleep habits and establish clear boundaries around mobile phone usage before bedtime may help reduce bedtime procrastination and improve sleep quality [48]. Similarly, interventions that address FOMO and promote healthier patterns of mobile phone use may help reduce the negative impact of mobile phone use on sleep quality [44].

Several limitations of this study should be acknowledged. First, the study used self-report measures, which are subject to biases and may not accurately reflect participants’ actual behaviors. Future studies could use objective measures, such as sleep monitoring devices or mobile phone usage trackers, to obtain more accurate data. Second, the study used a cross-sectional design, which precludes any causal conclusions. Future studies could use a longitudinal design to examine the causal relationships between mobile phone dependency, bedtime procrastination, FoMO, and sleep quality over time. Third, the data were collected during the campus lockdown period which may affect college students’ mobile phone usage and sleep the results should be interpreted with caution.

## Conclusion

The present study provided the evidence for the negative impact of mobile phone dependency on sleep quality in college students during the COVID-19 outbreak. The findings suggest that bedtime procrastination and FoMO may play a mediating role in this relationship. Therefore, interventions targeting bedtime procrastination and FOMO may be effective in improving sleep quality in college students who are dependent on their mobile phones. Additionally, the findings highlight the importance of promoting healthy mobile phone use habits and establishing boundaries around mobile phone usage to mitigate negative consequences on sleep quality.

## Data Availability

The datasets used and/or analyzed during the current study available from the corresponding author on reasonable request.
